# Deposition of Durable Micro Copper Patterns into Glass by Combining Laser-Induced Backside Wet Etching and Laser-Induced Chemical Liquid Phase Deposition Methods

**DOI:** 10.3390/ma13132977

**Published:** 2020-07-03

**Authors:** Jae Min Seo, Kui-Kam Kwon, Ki Young Song, Chong Nam Chu, Sung-Hoon Ahn

**Affiliations:** 1 Department of Mechanical & Aerospace Engineering, Seoul National University, 1, Kwanak-ro, Kwanak-gu, Seoul 08826, Korea; lovesmaller@snu.ac.kr (J.M.S.); kwonkuikam@snu.ac.kr (K.-K.K.); cnchu@snu.ac.kr (C.N.C.); 2 School of Mechanical Engineering, Soongsil University, 369, Sangdo-ro, Dongjak-gu, Seoul 06978, Korea; prema@snu.ac.kr

**Keywords:** laser induced chemical liquid phase deposition, laser-induced backside wet etching, additive machining, embedded metallic patterns on glass, anchor effect

## Abstract

Glass is a well-known non-conductive material that has many useful properties, and considerable research has been conducted into making circuits on glass. Many deposition techniques have been studied, and laser-induced chemical liquid phase deposition (LCLD) is a well-known and cost-effective method for rapid prototyping of copper deposition on glass. However, the deposition results from the LCLD method on the surface of glass, which shows an issue in its detachment from the substrates because of the relatively low adhesion between deposited copper and the nontreated glass surface. This problem undermines the usability of deposited glass in industrial applications. In this study, the laser-induced backside wet etching (LIBWE) method was performed as a preceding process to fabricate microchannels, which were filled with copper by LCLD. Additional durable copper wire was produced as a result of the enhanced adhesion between the glass and the deposited copper. The adhesion was enhanced by a rough surface and metal layer, which are characteristics of LIBWE machining. Furthermore, the proposed method is expected to broaden the use of deposited glass in industrial applications, such as in stacked or covered multilayer structures with built-in copper wires, because the inserted copper can be physically protected by the microstructures.

## 1. Introduction

Various deposition techniques on different materials, such as metallic melts [1], spray deposition [2], and chemical vapor deposition [3] have been widely studied over time. Among many target materials, interest in the industrial uses of glass has spread widely because glass possesses many useful properties, such as transparency and, chemical and physical stability. Microfluidic devices, sensors, smart glasses, microelectronics, and components of micromechanics are possible applications if glass possesses conductivity [4,5,6,7]. Several methods have been studied for combining conductive materials with glass, such as using indium tin oxide (ITO) [8,9], femtosecond laser deposition [10,11], electroless copper plating [12,13,14,15], and laser-induced chemical liquid phase deposition (LCLD) [16,17,18,19].

ITO technology involves selectively removing sputtered ITO from the surface of glass by wet etching or with a laser device [8], and femtosecond laser deposition is a selective electroless deposition method using femtosecond lasers on light-reactive glass [11], but these techniques require complex processes, are limited in substrate selection, and have higher costs than other methods. Electroless copper plating and LCLD are therefore more widely used because of process simplicity and cost-effectiveness [16]. 

Electroless copper plating is a non-electrolytic deposition method that creates a copper redox reaction by heating a deposition solution [12]. This method suffers from a lack of selectivity because the generated copper attaches to all over substrates. To selectively deposit copper to a desired area, prior treatment, such as creating a seed layer, is required before the deposition process [14]. A relatively long period of time is also needed to achieve sufficient height of copper using a plating method—15 h for a 25 µm height [12,13]. Furthermore, as the selectively deposited copper is not necessarily durable, a method to enhance the robustness of the copper wires is needed, because it would otherwise undermine its industrial utility. 

By comparison, small-sized metal deposition can be achieved in a selected area without a photomask or pre-treatment using the LCLD method [20], and less time is required for thicker copper deposition [21]. LCLD is, therefore, an easy, simple way to make copper circuits on glass, but the copper deposited by the LCLD method has an issue with durability because of its weak adhesion with glass. Although the LCLD method has been studied for several years, few studies were found on how to ensure the durability of deposited copper on a glass material [18,19].

Increasing the adhesion between glass and deposited copper could be a solution to ensuring the durability of copper deposited on glass using LCLD, and protecting the deposited copper physically by inserting copper into microstructures is another option [22]. Laser-induced backside wet etching (LIBWE) can form microstructures with rough surfaces and a metal layer, which could enhance adhesion between copper grains and glass [14,15]. These fabricated microstructures could also offer physical protection for installed copper wires.

In this study, LIBWE and LCLD were chosen to embed copper inside channels. Prior to conducting copper deposition, various sizes of channel were fabricated using the LIBWE method, and the dimensions and surface properties of the channels were analyzed. After the LIBWE process, LCLD was used to fill each channel with copper. The durability of copper wires, deposited on the surface of the glass and inside the channels, was measured. Resistance was measured and resistivities calculated to substantiate the potential for the copper to function as a circuit. Finally, an embedded copper circuit was created and tested as a simple application, and the potential for organizing a multilayer structure or stacking application was observed.

## 2. Principles 

### 2.1. Laser-Induced Backside Wet Etching

LIBWE is an easy and rapid way to fabricate various dimensions of microstructure on glass, regardless of its physical properties [23,24]. LIBWE is a technique to machine the backside of transparent glass by exploding absorbents using a laser beam [25]. Glass cannot be directly machined by lasers because it cannot absorb the 1064 nm wavelength of laser beams due to its transparency, but an absorbent that can absorb a 1064 nm wavelength can machine the glass indirectly. 

**Figure 1:**
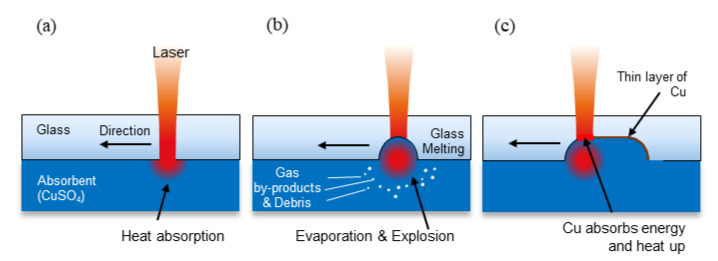
Brief mechanism of the LIBWE process: (**a**) An irradiated laser beam is absorbed by the absorbents. (**b**) Glass is removed by the heated absorbents. (**c**) Glass is continuously removed by the heated metal layer and absorbents.

A brief overview of the mechanism of LIBWE is shown in Figure 1. The laser beam energy heats up the absorbent at the interface of the absorbent and the glass (Figure 1a); the absorbance of the absorbent therefore match the wavelength of the laser beam. Ni- and Cu-based solutions are widely used as absorbents for 1064 nm wavelength laser devices [7,14]; for Cu-based solutions, CuSO_4_ is normally used for machining deeper channels and is thermally decomposed to gas-phase by-products, as shown in Equation (1) [24,26]:

3CuSO_4_ + Heat → 3CuO + SO_3_↑+ 2SO_2_↑+ O_2_↑ (1)

2Cu^2+^ + 2SO_2_ → Cu_2_O + 2SO_4_^2−^ (2)

During the LIBWE process, the temperature of the substrate is known to rise to approximately 1400 K, which is high enough to process the glass substrate [26]. As shown in Figure 1b, gas-phase by-products generated by the explosion of absorbents remove molten glass from the surface of the glass. A rough surface is formed over the machined area during the process of eliminating the molten glass particles. The SO_2_ generated, as shown in Equation (1), participates in a redox reaction with copper ions, shown in Equation (2) [26]. The Cu_2_O generated is deposited on the machined surface as a thin layer of copper compound. As laser energy constantly heats up the absorbent and deposited copper, glass is repeatedly removed from the glass substrate until reaching the goal depth [24]. LIBWE could therefore function as a pre-treatment process for glass because the created rough surface can offer anchor effect and the thin deposited layer of metal can offer additional adhesion force for further copper deposition [27,28,29].

### 2.2. Laser-Induced Chemical Liquid Phase Deposition

Copper deposition methods using a solution are based on the reduction reaction of copper ions. The chemical reaction of electroless copper deposition is as follows [30]:

[Cu(L)_2_]^2−^ + 2HCOH + 4OH^−^ + Energy → Cu^0^ + 2L^2−^ + H_2_ + H_2_O + 2HCOO^−^ (3)

As shown in Equation (3), the LCLD method needs a solution composed of four major elements—copper ions, a ligand (L), a reducing agent (HCOH), and OH^−^ ions. CuCl_2_ and CuSO_4_ are possible copper compounds for the copper ion supplementation. The resistance, topology, and shape of the generated copper wires depend on the type of copper compound used, and a CuCl_2_-based solution shows better topology than a CuSO_4_ solution [17,19]. A concentration of 0.05 M of Cu ions is appropriate because a reckless copper ion reduction reaction can occur at higher concentrations [19]. When the dissolved copper ions meets a reducing agent, such as formaldehyde (HCOH), a copper redox reaction may occur uncontrollably [31]; to prevent reckless and uncontrollable redox reactions, ligands need to be added to function as stabilizers for the copper ions [30], and potassium sodium tartrate tetrahydrate (KNaC_4_H_4_O_6_·4H_2_O; Rochelle salt) and ethylenediaminetetraacetic acid (C_10_H_16_N_2_O_8_; EDTA) are well-known ligands [19]. Lastly, the copper reduction reaction can occur in a high-pH alkaline surrounding, and the pH is controlled by NaOH in many cases [18].

An energy supply is needed to activate the copper reduction reaction. Applying heat by boiling the solution at around 350 K for several minutes supplies enough energy for copper redox reactions [15,32]. This boiling process can be replaced by a laser because the solution can be heated to sufficient temperature which can activate reduction reaction of copper ions in a short period of time [21]. Furthermore, the redox reaction of copper can occur in a more selected and focused area because the laser can heat in a more concentrated area than other heating methods.

**Figure 2:**
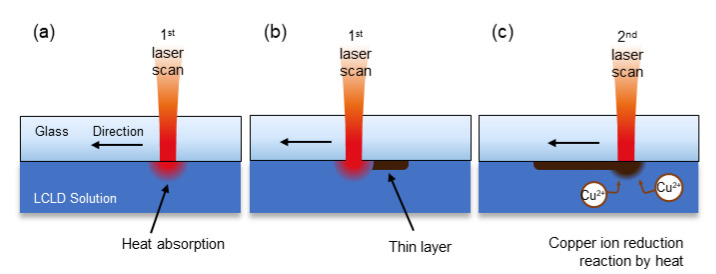
Brief mechanism of the LCLD process. (**a**) The laser beam is absorbed by the solution. (**b**) The copper ion reduction reaction occurs, and a thin layer of copper attaches to the surface of the glass. (**c**) Copper grows from the solution.

An overview of the mechanism of LCLD is shown in Figure 2. At the interface of the solution and the glass, the solution absorbs the laser beam energy (Figure 2a). As the energy of the laser beam, a copper reduction reaction occurs, and a thin copper layer attaches to the backside surface of the glass (Figure 2b). As the laser continues to supply heat energy to the LCLD solution, additional copper grows from the existing copper layer (Figure 2c).

## 3. Materials and Methods

### 3.1. Experimental Devices and Materials

**Figure 3:**
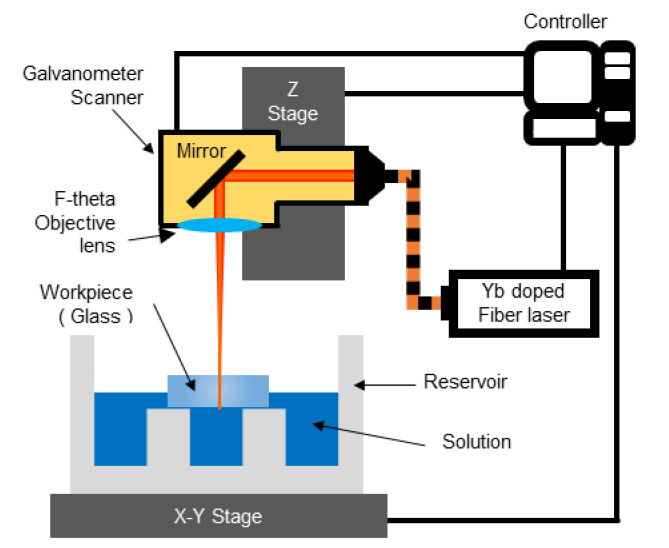
Schematic diagram of the LIBWE and LCLD systems.

A schematic diagram for the LIBWE and LCLD systems is shown in Figure 3. LIBWE and LCLD complement each other well, due to similarities in the experimental systems, laser devices, and related Cu-based solutions [7]. A 1064 nm wavelength Ytterbium pulsed fiber laser (YLP-C-1-100-20-20, IPG, Burbach, Germany) was used for both the LIBWE and LCLD processes. The laser has a spot size of 40 µm, pulse duration of 100 ns, and maximum power of 20 W. The peak irradiance of the laser beam was able to be set to 721.3 MW/cm^2^ through the source under the condition of a 20 kHz repetition rate of the pulsed laser and up to 180.3 MW/cm^2^ under the condition of 80 kHz repetition rate.

The focus of laser could be adjusted by moving the Z stage, because the galvanometer (SCANcube® 10, Scanlab, Puchheim, Germany) and an F-theta lens with a focal length of 160 mm were attached to it. The laser beam with a specified scanning speed was irradiated through the galvanometer, and the laser scan path was controlled by an RTC4 control board, while the galvanometer scanner traced a path over the materials.

Polycarbonate (PC) was used for the reservoir, which was fixed to the X-Y stage, as it does not react with either the LIBWE or LCLD solutions. 40 × 20 × 0.5 mm^3^ of soda-lime glass (JMC Glass, Ansan, Korea) was used as a substrate, which was fixed to a support of the reservoir to prevent it from moving. The position of the reservoir and the attached material was controlled by the X-Y stage. The X-Y and Z stages could be separately controlled to a 0.1 µm resolution using a motion controller. 

A three-dimensional (3D) surface profiler (μSurf, NanoFocus, Oberhausen, Germany) was used to analyze the maximum height of the profile (Rz) and the arithmetic mean surface roughness (Ra) of the fabricated LIBWE channels. The surface roughness of the channels was determined by averaging five measurements of the Ra and Rz along the channel direction. A scanning electron microscope (SEM) (JSM-6360, JEOL, Akishima, Japan) and energy-dispersive X-ray spectroscopy (EDS) (MERLIN Compact, Zeiss, Oberkochen, Germany) were used to detect the copper layer on the surface of the channels.

After completing the LIBWE and LCLD processes, cross-sections of the channels were prepared by cutting the workpiece. An ultrasonic transducer (DH.WUC.A02H, Daihan-Sci, Wonju, Korea) was used to check the durability of the deposited copper. Lastly, the conductivities were measured by measuring the resistance of 2 mm lengths of various dimensions of deposited copper using a multimeter (LCR HiTESTER 3511-50, HIOKI, Ueda, Japan).

### 3.2. Experimental Procedures

**Figure 4:**
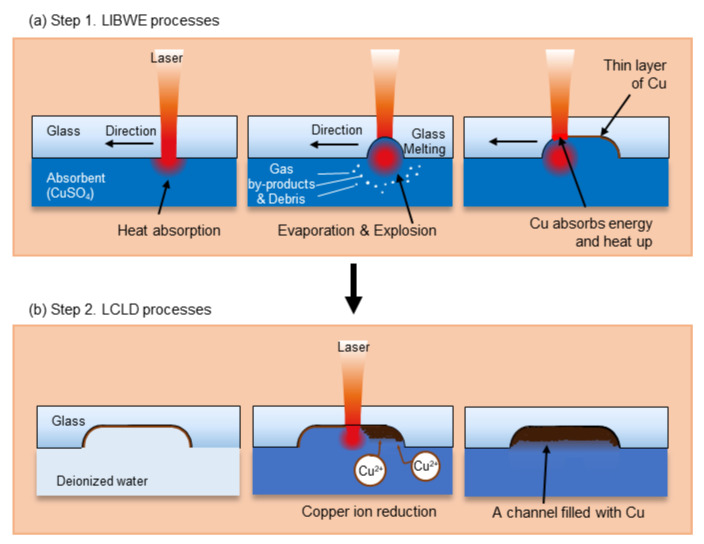
Sequence of the overall experiment. (**a**) LIBWE is applied as the first step. (**b**) LCLD is applied as the second step.

The overall experimental procedure can be explained in two steps (Figure 4). Step 1 was fabrication of microstructures by LIBWE, step 2 was filling the channels with copper by LCLD.

Before starting LIBWE, the reservoir was filled with absorbent liquid until the solution contacted the glass surface (Figure 4a), 0.7 M Copper (II) sulfate pentahydrate solution was used instead of a Ni-based solution to obtain a thin layer of copper, as mentioned in Section 2.1 [24,26].

After LIBWE, the reservoir was emptied and rinsed with deionized water (Figure 4b). As mentioned in Section 2.2, 0.05 M CuCl_2_ was chosen for LCLD for its better topology of deposited copper [19]. Rochelle salt at 0.1 M, was chosen as the ligand because it dissolves better in water than EDTA. While, 2 M formaldehyde was chosen for the reducing agent, and the pH was maintained at 12 by the concentration of NaOH during the LCLD process.

## 4. Results and Discussion

### 4.1. Fabrication of Channels by LIBWE 

Prior to conducting the deposition process, various dimensions of microchannels were fabricated by LIBWE with a 1064 nm pulsed laser. The LIBWE method is one way to fabricate microchannels with a rough surface, which creates an anchoring effect with deposited copper [11]. The laser parameters used for LIBWE are listed in Table 1.

**Figure 5:**
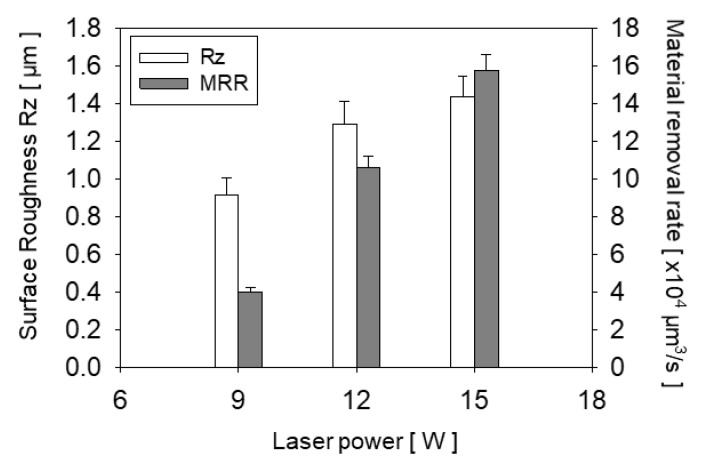
Graph of surface roughness and material removal rate for each laser power.

The surface roughness of each channel that was fabricated under a different laser power was measured and is shown in Figure 5 as a white bar. The result of 18 W is not presented because it generated cracks on glass and was thus not usable. While rough surfaces were obtained under every power of laser, the surface roughness increased when the laser power increased. Since a rougher surface shows a better adhesion force with deposited copper, 12 W and 15 W of laser power seem suitable for further experiments.

We selected 15 W of laser power because it showed a higher material removal rate (MRR) than 12 W, as shown in Figure 5 as a dark gray bar. The MRR difference between laser power affected the number of repeated laser scans when the goal depths of the channels were set at 50 µm to ensure sufficient embedding of the copper. To obtain the goal depth, 2000 repeated laser scans were needed under 9 W of laser power, 800 times at 12W, and 500 times at 15 W. As a result, 15 W of laser power was selected because a rough surface with high Rz could be obtained for anchoring and because it had a higher MRR.

**Figure 6:**
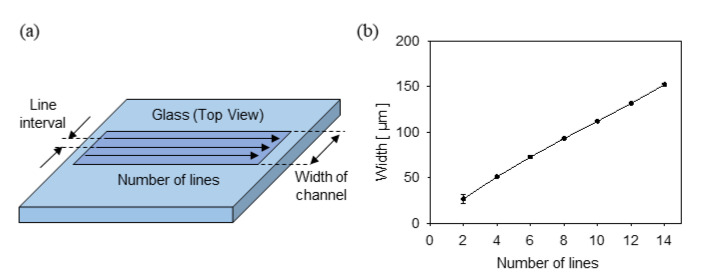
Controlling the width of the channels by the number of lines under LIBWE. (**a**) Definition of the number of lines and the line interval. (**b**) Fabricated channel widths according to the number of lines.

**Figure 7:**
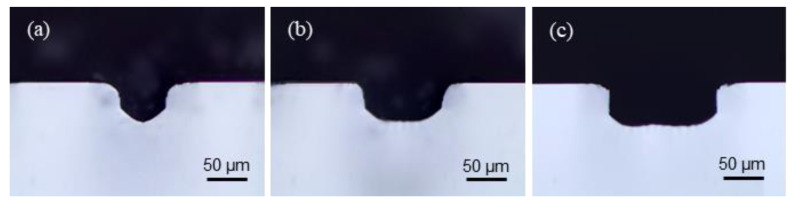
Optical images of fabricated channels from the section views at widths of (**a**) 50 µm, (**b**) 100 µm, and (**c**) 150 µm. Depths are 50 µm.

The widths of the microstructures were controlled by changing the number of lines at fixed 10 µm line intervals (Figure 6a); the relationship between the numbers of lines and the widths is shown in Figure 6b, displaying a linear relationship. Four, 9, and 14 lines were selected to obtain channel widths of 50 µm, 100 µm, and 150 µm, respectively. To confirm the dimensions of the fabricated channels, cross-sectional views were observed like shown in Figure 7. Various widths of channels with constant depth (50 µm) were prepared for the next step of LCLD process.

### 4.2. Laser Copper Deposition Inside of Channels

After fabrication of the micro channels, copper was deposited inside them by LCLD. The laser device parameters used for LCLD are listed in Table 2. The laser scan speed and repetition rate of the pulsed laser were fixed, and only the laser power and number of laser scans were varied.

**Figure 8:**
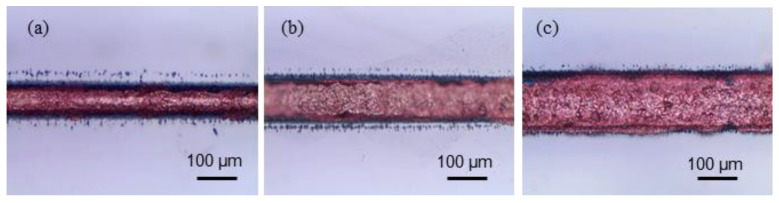
Optical images of embedded copper wires from the top view. Widths of (**a**) 50 µm, (**b**) 100 µm, and (**c**) 150 µm.

A method for adjusting the width of the deposited copper was needed as the widths of the channels were different. Unlike LIBWE, the width of deposited copper was not controlled by the number of lines (see Figure 6a). Only a single line was used because the width of the deposited copper could be adjusted simply by changing the laser power. As shown in Figure 8, the different widths of channel were filled with copper, without overflowing them, by changing the laser power; 2 W was sufficient to fill the 50 µm channel (Figure 8a), 3 W for 100 µm channel (Figure 8b), and 4 W was used to fill the 150 µm channel (Figure 8c). Using higher powers caused the copper to exceed the channels.

**Figure 9:**
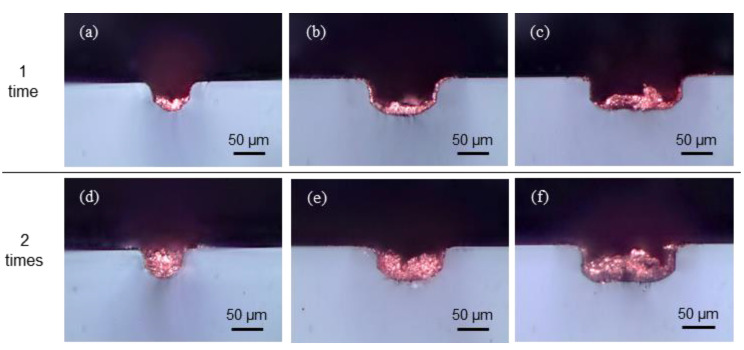
Optical images of embedded copper wires from a section view with a single laser scan and widths of (**a**) 50 µm, (**b**) 100 µm, and (**c**) 150 µm and with multiple laser scans and widths of (**d**) 50 µm, (**e**) 100 µm, and (**f**) 150 µm.

From the top view, copper appeared to fill the channels (Figure 8), and the actual filled ratio of copper was checked by preparing cross-sections (Figure 9). Cross-sectional views of the copper deposited with a single laser scan for the different widths are shown in Figure 9a–c. The deposited copper attached from the bottom, but the channels were not fully filled. A single laser scan was not sufficient to fully fill the channels, and because the height of the copper increases with additional number of scans were used. As shown in Figure 9d–f, each width of channel was fully filled with copper by two times of scans.

### 4.3. Durability of Deposited Copper

The durability of the copper wires was tested using an ultrasonic vibration transducer in deionized water under condition of 50 W and 28 kHz for 5 min. To compare the durability of the same dimensions of copper wire embedded in and on the surface of the glass, copper wires were tested with dimensions of 50 µm width and 30 µm height; these dimensions were chosen because of the difficulty of depositing more than 30 µm of height on the surface.

**Figure 10:**
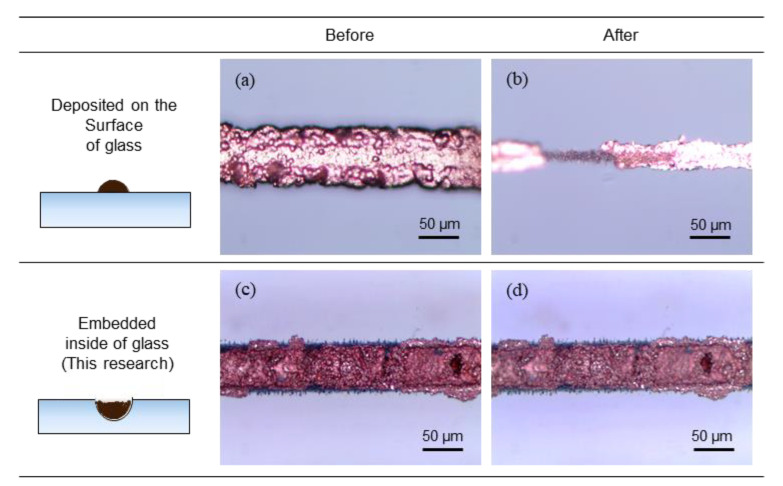
Durability test for deposited copper on the surface of the glass (**a**) before and (**b**) after the ultrasonic vibration test and inside channels; (**c**) before; and (**d**) after the ultrasonic vibration test.

Figure 10a,b show the copper wires on the surface of the glass being detached by ultrasonic vibration. As there were no prior treatments, such as making the surface rough by LIBWE, the adhesion between the glass surface and the deposited copper wire was relatively weak [14,28]. If the copper wire easily detaches from the substrates, as in this experiment, it would be more likely to fail in industrial applications. When the copper wire was embedded and installed inside a channel, more robust adhesion between the glass and copper was achieved. The results for the installed copper wire are shown in Figure 10c,d. Even after the ultrasonic vibration, the installed copper wire remained, proving more durable against external influences than copper wire on the surface.

**Figure 11:**
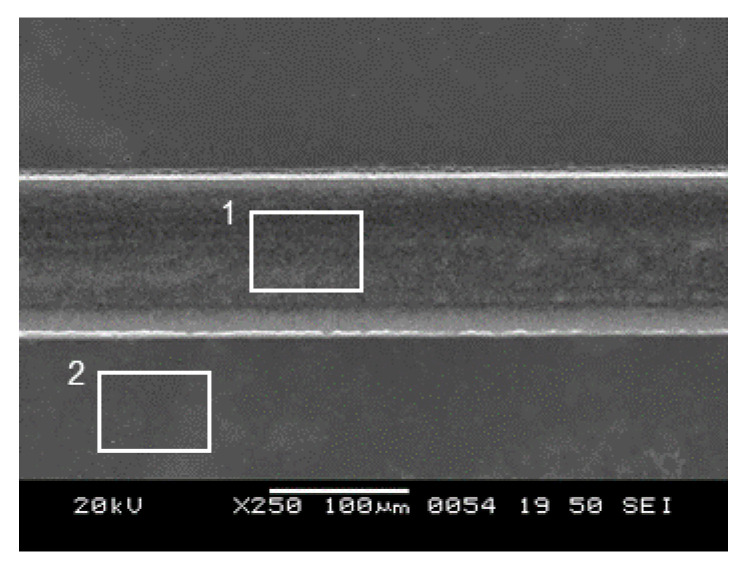
Analysis was conducted of the square areas outlined in white. Square 1 indicates the bottom surface of a channel and square 2 indicates the glass surface.

To analyze the reasons for the robustness of the embedded copper wire, a 3D profiler and EDS were used to measure the surface roughness (Rz and Ra) and detect copper on the surface. The results are shown in squares 1 and 2 in Figure 11. Square 1 indicates the inside of the channel, while, square 2 indicates the surface of the glass.

As shown in Table 3, the surface roughness of the bottom of the channel along the channel direction was 0.30 µm and of the glass surface was 0.01 µm in Ra value. As previously noted, a copper metal layer and sufficient surface roughness enhance the deposited copper’s durability with an anchor effect [27,28,29]. Based on previous research, the anchor effect, caused by roughness, is confirmed when the Ra exceeds 0.1 µm [14]. Since the Ra of the fabricated glass was 0.30 µm, the deposited copper persisted well in the vibration test, while the copper detached from the surface of the glass, which had an Ra of 0.01 µm. Even when a rough surface has been created, copper can still detach from the glass under ultrasonic vibration if the surface does not have a metal layer [14]. Hence, the detection of metal is needed to conclude an anchor effect, and copper was detected at the surface of the fabricated channel as shown in Table 3. Consequently, the durability of the copper wires was ensured by the rough surface, metal layer, and physical structure.

### 4.4. Conductivity and Applications

**Figure 12:**
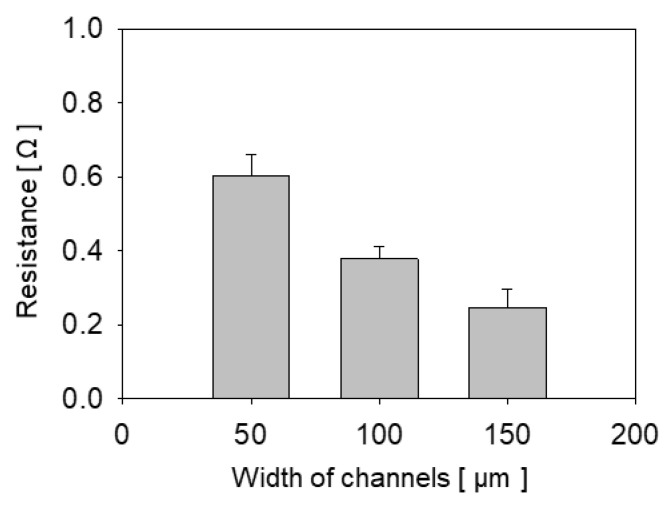
Graph of measured resistance for each width of deposited copper wire inside a channel.

Resistances were measured and resistivity calculated to check the conductivity of the inserted copper wires. The resistances of 2 mm lengths of each embedded copper wire were measured using a multimeter and shown in Figure 12. The amount of deposited copper increased with the widths of the channels, as shown in Figure 9d–f above, and the resistances decreased with increasing widths. 

Average resistivity of 87.52 µΩ·cm was calculated based on the assumptions that 50 µm thick uniform copper filled the channels that were rectangular with depths of 50 µm and widths of 50–150 µm. In previous research, resistivity using laser deposition was reported in a range of 10 to 300 µΩ·cm [7,18]. The range of the reference resistivity is relatively wide because resistivity varies significantly by the pH of the solution, the applied power, and the source of heat [18]. The resistivity achieved in this research was in the range of reference values and appears reasonable. Copper wire with reasonable resistivity can, therefore, be obtained from the combined processes described herein.

**Figure 13:**
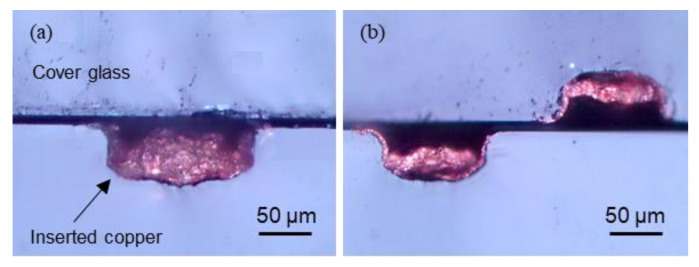
Cross section images of (**a**) a fully filled 150 µm wide channel sealed with cover glass; and (**b**) two layers of half-filled 100 µm wide channels stacked without supports.

More applications can be considered when copper is installed inside a channel. The entrance of the channel can be sealed with cover glass or other materials to improve usability and security, such as shown in Figure 13a. By doing this, more durable copper wire can be achieved because it is physically protected by the micro channels and glass covering from external conditions. Since many techniques for bonding glass and other materials have been introduced, it seems that attaching cover glass to the first layer of deposited glass should be possible. The femtosecond laser direct welding technique is one good possible option for bonding glass with covering materials because of its simplicity and short process time [33]. Furthermore, as shown in Figure 13b, copper wires can also be organized in several layers without the installation of supports. If channel deposition can be combined with via-hole deposition, it appears possible to organize layer-to-layer conductive circuits. [34].

**Figure 14:**
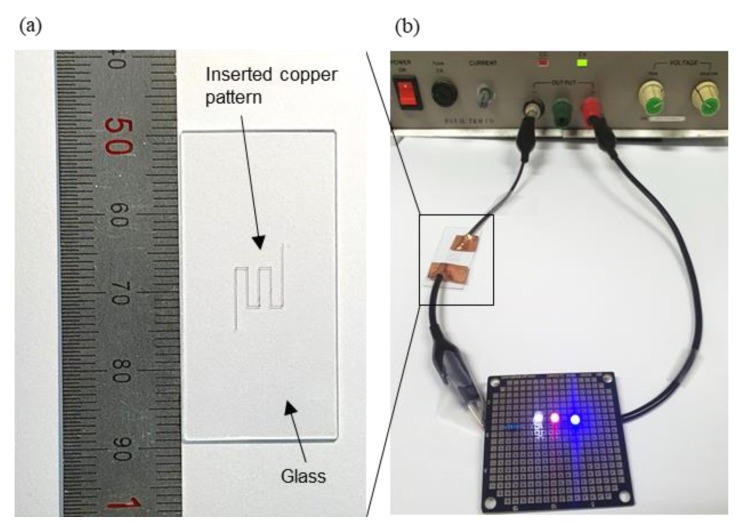
Conductivity test of embedded copper wire. (**a**) Shape of the embedded copper pattern. (**b**) Simple LED circuit.

To assess the functionality of a copper circuit obtained from the combined process, 37 mm of a zig-zag embedded pattern was prepared, as shown in Figure 14a. The embedded pattern was at a depth of 50 µm with a width of 100 µm. The resistance of the pattern was 7.3 Ω and a simple LED circuit was found to fully function when 9 V was applied, as shown in Figure 14b. It was, thus, confirmed that the combined process can form fully functioning copper circuit patterns inside the microstructures.

## 5. Conclusions

LCLD is a widely used deposition method because of its simplicity and cost-effectiveness, using relatively inexpensive laser devices for selective area and small-sized metal deposition. Despite the many advantages of LCLD, easy detachment issues have not been actively discussed. Adhesion between deposited copper and glass is weak, and increasing the adhesion force would be helpful in expanding the uses of deposited glass. In this study, copper wire was successfully installed inside channels by combining LIBWE and LCLD. Channels were precisely fabricated with LIBWE using a near-infrared laser source and absorbents. After replacing the solution with that for the LCLD process, copper deposition was conducted sequentially using the same equipment, and after sufficient number of laser scans, the channels became filled with copper. 

With LIBWE surface treatment, a thin copper layer and rough surface were formed on the soda-lime glass. By using comparative tests with embedded copper and surface deposited copper, it was found that the initially formed copper layer and the surface roughness are the major factors in increasing the attachment of the copper deposition. The measured resistance varies depending on the amount of copper, and the calculated resistivity was 87.52 µΩ·cm. The resistivity achieved in this research can be considered reasonable in light of previous research. An embedded form of copper deposition, stacking or sealing could increase possible applications yet further. It was also confirmed that a configured built-in copper pattern was fully operational.

## Figures and Tables

**Table 1:** Laser parameters for LIBWE.

Variables	Values
Laser power [W]	9, 12, 15, 18
Scan speed [mm/s]	150
Repetition rate of pulsed laser [kHz]	50
Scan length [mm]	2
Number of repeated laser scan	500, 800, 2000
Line interval [µm]	10
Number of lines	4, 9, 14

**Table 2:** Laser parameters for LCLD.

Variables	Values
Laser power [W]	2, 3, 4
Scan speed [mm/s]	0.015
Repetition rate of pulsed laser [kHz]	80
Scan length [mm]	2
Number of laser scan	1, 2

**Table 3:** Surface roughness and detection of Cu for each area of glass analyzed.

Area	Measured Surface Roughness		Area	Detection of Cu
	Rz (µm)	Ra (µm)		
Bottom of channel	1.44	0.30	Square 1	O
Surface of glass	0.05	0.01	Square 2	X

## References

[1] Moqadam S.I., Mädler L., Ellendt N. (2019). A high temperature drop-on-demand droplet generator for metallic melts. Micromachines.

[2] Wu Y., Du J., Choy K.L., Hench L.L. (2007). Laser densification of alumina powder beds generated using aerosol assisted spray deposition. J. Eur. Ceram. Soc..

[3] Quinto D.T., Wolfe G.J., Jindal P.C. (1987). High temperature microhardness of hard coatings produced by physical and chemical vapor deposition. Thin Solid Films.

[4] Crawford W.J. (2005). Verb agreement and disagreement. J. Engl. Linguist..

[5] Han M.J., Khang D.Y. (2015). Glass and plastics platforms for foldable electronics and displays. Adv. Mater..

[6] Kim J.G., Nam K.G., Cho S.H., Chang W.S., Na S.J., Whang K.H. (2006). Micromachining characteristics inside transparent materials using femtosecond laser pulses. J. Korean Soc. Precis. Eng..

[7] Zehnder S., Schwaller P., von Arx U., Neuenschwander B. Laser-induced chemical liquid-phase deposition of copper on transparent substrates. Proceedings of the International Conference on Advanced Laser Technologies.

[8] Chen M.F., Chen Y.P., Hsiao W.T., Gu Z.P. (2007). Laser direct write patterning technique of indium tin oxide film. Thin Solid Films.

[9] Wang M.W., Liu T.Y., Pang D.C., Hung J.C., Tseng C.C. (2014). Inkjet printing of a PH sensitive palladium catalyst patterns of ITO glass for electroless copper. Surf. Coat. Technol..

[10] Sugioka K., Masuda M., Hongo T., Cheng Y., Shihoyama K., Midorikawa K. (2004). Three-dimensional microfluidic structure embedded in photostructurable glass by femtosecond laser for lab-on-chip applications. Appl. Phys. A Mater. Sci. Process..

[11] Hanada Y., Sugioka K., Midorikawa K. (2008). Selective metallization of photostructurable glass by femtosecond laser direct writing for biochip application. Appl. Phys. A Mater. Sci. Process..

[12] Deckert C.A. (1995). Electroless copper plating. A review: Part I. Plat. Surf. Finish..

[13] Honma H., Kobayashi T. (1994). Electroless copper deposition process using glyoxylic acid as a reducing agent. J. Electrochem. Soc..

[14] Kim H.G., Park M.S. (2017). Circuit patterning using laser on transparent material. Surf. Coat. Technol..

[15] Long J., Li J., Li M., Xie X. (2019). Fabrication of robust metallic micropatterns on glass surfaces by selective metallization in laser-induced porous surface structures. Surf. Coat. Technol..

[16] Kordas K., Bali K., Nanai L., Galbacs G., Uusimaki A., Leppavuori S. (2000). Reaction dynamics of CW ar laser induced copper direct writing from liquid electrolyte on polyimide substrates. Appl. Surf. Sci..

[17] Manshina A.A., Povolotskiy A.V., Ivanova T.Y., Tver’Yanovich Y.S., Tunik S.P., Kim D., Kim M., Kwon S.C. (2007). Effect of salt precursor on laser-assisted copper deposition. Appl. Phys. A Mater. Sci. Process..

[18] Chen Q.J., Imen K., Allen S.D. (2000). Laser enhanced electroless plating of micron-scale copper wires. J. Electrochem. Soc..

[19] Kochemirovsky V.A., Skripkin M.Y., Tveryanovich Y.S., Mereshchenko A.S., Gorbunov A.O., Panov M.S., Tumkin I.I., Safonov S.V. (2015). Laser-induced copper deposition from aqueous and aqueous-organic solutions: State of the art and prospects of research. Russ. Chem. Rev..

[20] Tumkin I.I., Kochemirovsky V.A., Bal’makov M.D., Safonov S.V., Zhigley E.S., Logunov L.S., Shishkova E.V. (2015). Laser-induced deposition of nanostructured copper microwires on surfaces of composite materials. Surf. Coat. Technol..

[21] Wang X.C., Zheng H.Y., Lim G.C. (2002). Laser induced copper electroless plating on polyimide with Q-switch Nd:YAG laser. Appl. Surf. Sci..

[22] Seo J.M. (2020). Fabrication of Embedded Copper Wire on Glass by Laser-Induced Chemical Liquid Phase Deposition with Ytterbium Fiber Laser. Master’s Thesis.

[23] Kwon K.-K., Kim H., Kim T., Chu C.N. (2020). High Aspect ratio channel fabrication with near-infrared laser-induced backside wet etching. J. Mater. Process. Technol..

[24] Huang Z.Q., Hong M.H., Do T.B.M., Lin Q.Y. (2008). Laser etching of glass substrates by 1064 Nm Laser irradiation. Appl. Phys. A Mater. Sci. Process..

[25] Kawaguchi Y., Ding X., Narazaki A., Sato T., Niino H. (2005). Transient pressure induced by laser ablation of toluene, a highly laser-absorbing liquid. Appl. Phys. A Mater. Sci. Process..

[26] Xie X., Huang X., Jiang W., Wei X., Hu W., Ren Q. (2017). Three dimensional material removal model of laser-induced backside wet etching of sapphire substrate with CuSO4 solutions. Opt. Laser Technol..

[27] Bugaev S.P., Sochugov N.S. (2000). Production of large-area coatings on glasses and plastics. Surf. Coat. Technol..

[28] Chong E.K., Stevens M.G., Nissen K.E. (2003). Effect of surface roughness on the adhesion of electrolessly plated platinum to poly(ethylene terephthalate) films. J. Adhes..

[29] Okamoto N., Wang F., Watanabe T. (2004). Adhesion of electrodeposited copper, nickel and silver films on copper, nickel and silver substrates. Mater. Trans..

[30] Kochemirovsky V.A., Menchikov L.G., Kuz’Min A.G., Safonov S.V., Tumkin I.I., Tver’Yanovich Y.S. (2012). Side reactions during laser-induced deposition of copper from aqueous solutions of cuii complexes. Russ. Chem. Bull..

[31] Kochemirovsky V.A., Fateev S.A., Logunov L.S., Tumkin I.I., Safonov S.V. (2014). Laser-induced copper deposition with weak reducing agents. Int. J. Electrochem. Sci..

[32] Hanna F., Abdel Hamid Z., Abdel Aal A. (2004). Controlling factors affecting the stability and rate of electroless copper plating. Mater. Lett..

[33] Kim S., Kim J., Joung Y.H., Choi J., Koo C. (2018). Bonding strength of a glass microfluidic device fabricated by femtosecond laser micromachining and direct welding. Micromachines.

[34] Alkire R.C., Romankiw L.T. (1989). Plating of copper into through-holes and vias. J. Electrochem. Soc..

